# Muscle and fat matter: Automated CT-based body composition analysis predicts survival in *Hepatocellular carcinoma* patients undergoing radioembolization

**DOI:** 10.1016/j.ejro.2025.100721

**Published:** 2026-01-19

**Authors:** Hannah L. Steinberg-Vorhoff, Anneke Ketelsen, Tabea Schuch, Jens M. Theysohn, Benedikt M. Schaarschmidt, Johannes Haubold, Farroch Vahidi Noghani, Matthias Jeschke, Leonie Jochheim, Johannes M. Ludwig

**Affiliations:** a Department of Diagnostic and Interventional Radiology and Neuroradiology, University Hospital Essen, University of Duisburg-Essen, Hufelandstraße. 55, Essen 45147, Germany; b Department of Gynecology, University Hospital Hamburg Eppendorf, Martinistraße 52, Hamburg 20251, Germany; c Department of Dermatology and Allergology, Helios Klinikum Berlin-Buch, Schwanbecker Ch 50, Berlin 13125, Germany; d Department of Radiology and Interventional Radiology, University Hospital Bochum, Bürkle de la Camp-Platz 1, Bochum 44789, Germany; e Department of Radiology and Nuclear Medicine, University Medical Center Mannheim, Heidelberg University, Theodor-Kutzer-Ufer 1-3, Mannheim 68167, Germany; f Department of Gastroenterology and Hepatology, University Hospital Essen, University of Duisburg-Essen, Hufelandstr. 55, Essen 45147, Germany

**Keywords:** HCC, SIRT, Radioembolization, BCA

## Abstract

**Purpose:**

This study aimed to assess the prognostic significance of pretreatment CT-based body composition markers in patients with *Hepatocellular carcinoma* (HCC) treated with radioembolization.

**Material and methods:**

Automated analysis of baseline CT scans was performed to retrospectively evaluate body composition (BCA) parameters in 198 patients from a prospective registry database, including skeletal muscle (SM) and bone (B) volumes. BCA parameters and ratios were dichotomized using a maximally selected log-rank approach. Kaplan-Meier and uni- (UVA) and multivariate (MVA) Cox-proportional-hazard ratio (HR) survival analyses were performed.

**Results:**

The median survival time was 18.5 months. In UVA, lower BCLC stage, ≦ 70 years of age, normal serum albumin, non-elevated C-reactive protein, normal aspartate aminotransferase (ASAT), normal alkaline phosphatase, normal gamma-glutamyl transaminase (GGT), absence of portal vein thrombosis and various BCA parameters were statistically significant with the skeletal muscle to bone ratio (SM/B) demonstrating the strongest survival discrimination with a median survival of 23.6 months for high and 12.0 months for low SM/B (HR: 0.65, 95 %CI: 0.46–0.9; p = 0.0001). In MVA, SM/B, BCLC stage, ASAT, and GGT remained independently significant. Patients with higher SM/B ratios demonstrated a significantly higher disease control rate during the initial imaging follow-up after three months (74.4 % vs. 54.0 %, p = 0.017).

**Conclusion:**

These findings suggest that fully automated, CT-based measurement of BCA parameters — particularly the SM/B ratio — can serve as an independent prognostic factor for survival and disease control in patients with *Hepatocellular carcinoma* (HCC) undergoing radioembolization. This could potentially facilitate the identification of patients who would benefit most from this treatment.

## 1 Introduction

Liver cancer is the sixth most commonly diagnosed cancer and the third leading cause of cancer-related deaths, posing a significant public health challenge worldwide [1]. According to the Barcelona Clinic Liver Cancer (BCLC) Staging System, radioembolization remains a treatment option for a selected group of patients with *Hepatocellular carcinoma* (HCC) [2]. However, the variability in treatment outcomes following radioembolization underscores the need to identify reliable prognostic factors to determine the potential benefits of this treatment as part of the decision-making process when selecting the most suitable therapy for each patient individually.

In addition to well-established risk stratification factors, such as BCLC stage, liver function, tumor burden, and the proportions of muscle, fat, and bone, also referred to as body composition, these factors are increasingly recognized as potential markers for treatment selection and prognostication in patients with HCC [3], [4]. Among the various body composition markers, sarcopenia, defined as the loss of skeletal muscle mass, quality, and strength, is arguably one of the most extensively studied and established prognostic parameters in patients with cancer, with a pooled prevalence of approximately 39 % in patients with HCC [5], [6]. Sarcopenia and cancer cachexia are intricate conditions marked by progressive skeletal muscle loss and serve as significant indicators of reduced survival rates across various cancer types, including HCC [3], [4], [7], [8]. Sarcopenia can lead to diminished functional capacity and impaired ability to perform activities of daily living. It is frequently associated with metabolic disturbances, such as insulin resistance, vitamin D deficiency, and elevated inflammatory markers, all of which contribute to the progression of liver fibrosis and the development of *Hepatocellular carcinoma* (HCC) [8]. A comprehensive meta-analysis revealed a substantial impact of sarcopenia on the prognosis of *Hepatocellular carcinoma* (HCC), irrespective of the treatment modality, although data on radioembolization were limited [6], [9]. Similarly, an association between visceral adiposity and intramuscular fat deposition in the development of *Hepatocellular carcinoma* (HCC) has been demonstrated [3], [10], [11], [12], [13], [14]. Nevertheless, the prognostic significance of visceral adiposity remains equivocal, with some reports indicating a detrimental effect on overall survival, while others propose a protective effect, particularly in patients with advanced liver disease [3], [8], [12], [13], [14]. The prognostic value of intramuscular fat in patients with *Hepatocellular carcinoma* (HCC) has also been demonstrated, supporting its relevance in predicting overall mortality [8], [15]. Nevertheless, this association has not been observed in patients treated with radioembolization [16]. Despite the mounting evidence for body composition markers in general, data regarding their prognostic value in patients treated with radioembolization are limited and, in some cases, contradictory, with even negative results. [4], [9], [16], [17].

Historically, body composition analysis has relied on the manual segmentation of computed tomography (CT) images, a process that is both labor-intensive and impractical for routine clinical use. Recent advances in automated CT-based analysis, leveraging convolutional neural networks, have revolutionized this field by providing rapid, reproducible, and standardized measurements of the muscle, adipose tissue, and bone compartments of the entire abdominal cavity, and not only at the level of the third lumbar vertebra (L3), as commonly performed in most studies [3], [18], [19]. These tools have the potential to facilitate the incorporation of body composition assessment into clinical workflows, thereby enhancing prognostic stratification. This study aimed to assess the prognostic significance of pretreatment CT-based body composition markers in patients with HCC treated with radioembolization in terms of overall survival and treatment response.

## 2 Material and methods

A total of 654 patients with *Hepatocellular carcinoma* (HCC) were treated with radioembolization at our institution between 2010 and 2021 and included in a prospective registry database following informed consent. The censoring date for follow-up was October 15, 2023. The inclusion criteria were as follows: age ≥ 18 years, histological or imaging-based diagnosis of HCC according to the European Association for the Study of the Liver criteria [20], and an institutional tumor board decision recommending radioembolization. Of these, 198 patients had available baseline computed tomography (CT) images of the abdomen and were included in this study. Automated analysis of the CT scans was performed retrospectively. Informed consent was obtained from the Institutional Review Board (approval numbers 21–10097-BO and 13–5325-BO).

### 2.1 Radioembolization

Radioembolization was conducted using yttrium-90-loaded glass-based microspheres with a diameter of 20–30 µm (TheraSphere™, Boston Scientific, Massachusetts, USA). The efficacy of the treatment was evaluated using transarterial angiography, in which technetium-99 m macroaggregated albumin (MAA) was administered into the target liver volume(s). Subsequently, single-photon emission computed tomography (SPECT-CT) was performed to evaluate the flow and shunting to extrahepatic organs, including the determination of the lung shunt fraction. Treatments were planned using a single-compartment model, as previously described [21]. Radioembolization treatment was commonly performed once. However, in the case of whole liver treatment, the lobes were usually treated sequentially with 4–6 weeks between treatments. The median dose administered to the target volume was 117 Gy. Radioembolization was performed in patients who were not eligible for curative treatment (e.g., thermal ablation, resection, or liver transplantation, unless used for bridging to transplantation) and were not considered systemic therapy options by the multidisciplinary tumor board at our institution.

### 2.2 Automated body composition analysis

**Fig. 1:**
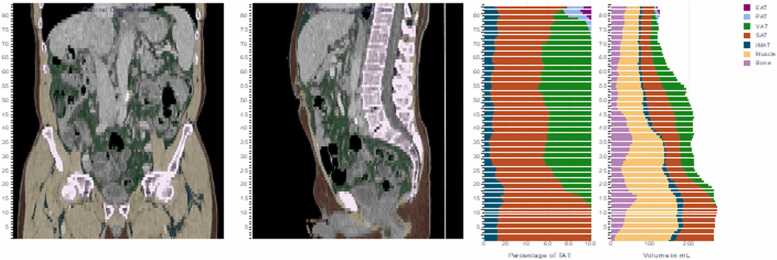
The example illustrates the fully automated 3D segmentation of an abdominal CT scan, with the various body composition volumes colored accordingly: total adipose tissue (TAT), visceral adipose tissue (VAT), subcutaneous adipose tissue (SAT), epicardial adipose tissue (EAT), pericardial adipose tissue (PAT), intra- and intermuscular adipose tissue (IMAT), skeletal muscle (SM), and bone.

The body composition analysis (BCA) parameters were evaluated using an advanced, fully automated, and open-source solution for CT data analysis [19], [22], [23], [24]. The software and source code can be obtained at https://github.com/UMEssen/Body-and-Organ-Analysis. The applied version was v0.1.18. This method employs a pre-trained convolutional neural network to perform quantitative 3D volumetry of the body tissue composition from regular abdominal CT scans. To ensure consistency across different acquisition slice thicknesses, the algorithm automatically resampled the data into 5 mm slices before analysis. This system was developed and validated using CT datasets with various acquisition parameters. The algorithm provided the following BCA parameters: total adipose tissue volume (TAT), visceral adipose tissue volume (VAT), subcutaneous adipose tissue volume (SAT), intra- and intermuscular adipose tissue volume (IMAT), skeletal muscle volume (SM), and bone volume (B) (Fig. 1). To accommodate discrepancies in patient dimensions and scanning areas, a normalization process was implemented for body composition parameters. This approach involves the automated selection of the abdominal cavity and division of the volume measurements by the number of detected slices within the abdominal cavity, thereby obtaining a mean standardized value for the aforementioned parameters. CT-scans included were usually obtained within a median 6.3 weeks (IQR: 4.9 – 9.1) prior to radioembolization.

### 2.3 Assessment of hepatic tumor response and survival

The response to treatment was evaluated according to the modified Response Evaluation Criteria in Solid Tumors (mRECIST) [25]. The time to progression (TTP) was calculated from the date of the initial radioembolization procedure. In instances where patients were lost to follow-up imaging, the date of the most recent imaging study was used as the censoring date.

### 2.4 Statistics

The median overall survival (OS) and time to progression (TTP) were calculated using Kaplan-Meier analysis, and the log-rank test was employed to assess the significance of these findings. For univariate (UVA) and multivariate (MVA) analyses, a Cox proportional hazard model was employed to calculate hazard ratios (HR) and 95 % confidence intervals (CI). However, in instances where there was substantial overlap or significant correlation among the factors, the strongest HR was selected for the MVA. Body composition parameters, indices, and ratios were dichotomized using a maximally selected log-rank approach. The 10th–90th percentile values (in 10 % steps) for each parameter were examined, and the cut-point that yielded the smallest two-sided log-rank p-value between the "low" (≤ cutoff) and "high" (> cutoff) groups was selected. In the case of statistically significant sex-related differences in body composition parameters, a sex-specific cutoff was applied (Tables S1 and S2). As the data were normally distributed, Student's *t*-test was applied to assess sex-related differences.

Laboratory values were dichotomized using sex-specific upper limits of normal. Contingency analysis was conducted using Pearson's correlation coefficient. The two groups were compared using the Mann-Whitney U-test, and more groups were compared using the ANOVA test. No alpha error correction was performed due to the exploratory nature of this study. Statistical significance was set at p < 0.05. Statistical analyses were performed using the JMP 18.2 software (SAS Institute Inc., Cary, NC, USA).

## 3 Results

### 3.1 Demographics

This study included 198 participants with a median age of 71 years (range: 20–89 years), of whom 22.7 % were female. In total, 46 patients were either censored or lost to follow-up, with a median follow-up period of 35.8 months (95 % CI: 31.9–46.3 months). The baseline characteristics of the study cohort are presented in Table 1.

**Table 1:** Baseline characteristics of the patients.

Baseline Characteristic	Number of patients (total n = 198)
Liver cirrhosis	148 (74.7 %)
Etiology of cirrhosis	
• Alcohol • Non-alcoholic steatohepatitis • Viral • Hemochromatosis • Other	39 (19.7 %) 35 (17.7 %) B:17 (8.6 %); C:35 (17.7 %) 7 (3.5 %) 15 (7.8 %)
Ascites	
• None • Mild	187 (94.4 %) 11 (5.6 %)
Disease extent	
• Bilobar • Unilobar	104 (52.5 %) 94 (47.5 %)
Portal vein thrombosis	
• main • lobar • segmental • None	1 (0.5 %) 13 (6.6 %) 16 (8.1 %) 168 (84.8 %)
Child-Pugh class	
• A • B	190 (96 %) 8 (4 %)
BCLC stage	
• A • B • C	29 (14.6 %) 120 (60.6 %) 49 (24.7 %)
ALBI grade	
• 1 • 2	161 (81.3 %) 37 (18.7 %)
Pretreatment • Resection • Chemoembolisation • Sorafenib • Thermal ablation • Other	67 (33.8 %) 35 (17.7 %) 22 (11.1 %) 11 (5.6 %) 5 (2.5 %) 4 (2 %)
Hepatic encephalopathy	
• Stage 1 • None	18 (9.1 %) 180 (90.9 %)

Baseline characteristics of the patients. Patients may have received more than one prior therapy. Abbreviations: BCLC (Barcelona Clinic Liver Cancer), ALBI (albumin-bilirubin).

### 3.2 Survival analysis

**Fig. 2:**
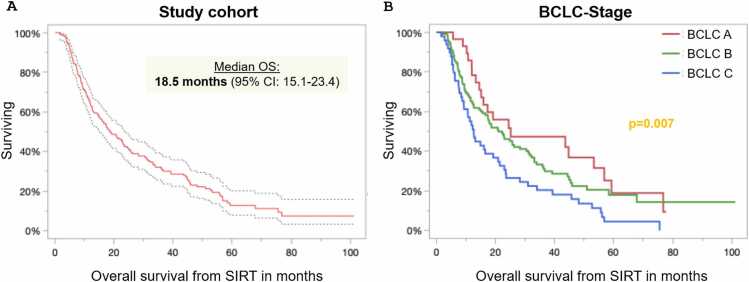
Overall survival of the study cohort (A) and stratified by BCLC stage (B) is shown in months from radioembolization. 95 %CI: 95 % confidence interval; BCLC: Barcelona Clinic Liver Cancer Classification.

The median survival time of the study cohort was 18.5 months (95 % CI, 15.1–23.4 months). The 6-month, 1-year, 2-year, 3-year, 4-year, and 5-year survival rates were 86.4 %, 62.6 %, 37.4 %, 24.2 %, 14.1 %, and 6.1 %, respectively. The median overall survival times according to BCLC stage A/B/C were 25.1 (95 % confidence interval [CI]: 15.1–56.8), 20.9 (95 % CI: 15.6–30.4), and 12.6 (95 % CI: 9.2–19.2), respectively (log-rank; p = 0.007) (Fig. 2).

In the univariate analysis, several pretreatment factors were found to be significantly associated with overall survival (Table 2). In this analysis, an age of up to 70 years, lower BCLC stage, presence of normal serum albumin, non-elevated C-reactive protein, normal aspartate aminotransferase (ASAT), normal alkaline phosphatase, normal gamma-glutamyl transaminase (GGT), and absence of portal vein thrombosis were significantly associated with survival benefits.

**Table 2:** Uni- and multivariate survival analyses of pretreatment factors.

				**Univariate analysis**		**Multivariate analysis**	
**Groups**		**Number of patients**	**Median overall survival in months (95 % CI)**	**HR (95 % CI)**	**p-value**	**HR (95 % CI)**	**p-value**
Gender	Female	45	15.9 (10.8 – 24.8)	1	0.38	-	-
	Male	153	20.9 (14.9 – 25.6)	0.84 (0.58 – 1.23)		-	
Age	> 70 years	100	14.5 (10.5 – 17.9)	1	**0.007**	1	0.09
	≦ 70 years	98	25.6 (18.5 – 34.0)	0.64 (0.47 – 0.85		0.72 (0.5 – 1.04)	
Child-Pugh class	A	190	18.5 (14.8 – 23.4)	1	0.74	-	-
	B	8	28.2 (4.9 – 45.7)	0.88 (0.41 – 1.87)		-	
BCLC stage*	A	29	25.1 (15.1 – 56.8)	0.48 (0.28 – 0.81)	**0.009**	0.21 (0.28 – 0.64)	**0.0009**
	B	120	20.9 (15.6 – 30.4)	0.63 (0.44 – 0.91)		0.74 (0.51 – 1.08)	
	C	49	12.6 (9.2 – 19.2)	1		1	
ALBI grade	1	161	20.9 (15.6 – 25.7)	0.68 (0.45 – 1.02)	0.072	-	-
	2	37	15.9 (6.9 – 21.4)	1		-	
Ascites	no	187	19.1 (15.1 – 23.6)	0.93 (0.47 – 1.84)	0.84	-	-
	yes	11	18.5 (9.6–56.1)	1		-	
Sum of hepatic mRECIST lesions	≦ median (4.5 cm)	104	22.95 (15.1 – 30.97)	0.84 (0.6 – 1.16)	0.35	-	-
	> median	94	17.4 (12.3 – 22.2)	1		-	
Portal vein thrombosis	no	168	22.6 (16.5 – 28.2)	0.52 (0.35 – 0.79)	**0.004**	-	-
	yes	30	12.4 (7.7 – 19.17)	1		-	
Involvement	unilobar	94	19.2 (12.7 – 31.1)	0.87 (0.63 – 1.2)	0.41	-	-
	bilobar	104	17.95 (13.28 – 22.95)	1		-	
CRP	not elevated	102	25.1 (19.1 – 39.2)	0.55 (0.4 – 0.76)	**0.0003**	0.79 (0.55 – 1.14)	0.2
	elevated	96	12.7 (15.1 – 23.4)	1		1	
Albumin	≦ 3.4 g/dl	7	9.9 (6.94 – 21.4)	1	**0.049**	0.52 (0.22 – 1.24)	0.18
	> 3.4 g/dl	191	19.2 (15.6 – 24.8)	0.39 (0.17 – 0.89)		1	
Bilirubin	≦ 1.2 mg/ml	174	19.1 (15.1 – 23.6)	1	0.72	-	-
	> 1.2 mg/ml	24	15.9 (7.4 – 36.3)	0.91 (0.53 – 1.55)		-	
Prior HCC therapy	no	129	19.1 (14.8 – 24.4)	0.99 (0.72 – 1.37)	0.96	-	-
	yes	69	18.5 (11.1 – 33.9)	1		-	
ASAT (GOT)**	≦ 35 (f) / 50 (m) U/L	97	32.2 (22.95 – 44.4)	0.52 (0.38 – 0.72)	**< 0.0001**	0.58 (0.4 – 0.83)	**0.0027**
	> 35 (f) / 50 (m) U/L	101	12.2 (9.9 – 15.9)	1		1	
ALAT (GPT)**	≦ 35 (f) / 50 (m) U/L	141	20.9 (15.6 – 28.2)	0.77 (0.55 – 1.08)	0.14	-	-
	> 35 (f) / 50 (m) U/L	57	15.9 (10.69 – 23.41)	1		-	
AP**	≦ 105 (f) / 130 (m) U/L	98	25.6 (22.19 – 35.9)	0.57 (0.41 – 0.78)	**0.0006**	0.94 (0.62 – 1.43)	0.77
	> 105 (f) / 130 (m) U/L	100	12.6 (10.65 – 16.47)	1		1	
GGT**	≦ 38 (f) / 55 (m) U/L	30	43.6 (33.14– 52.7)	0.5 (0.31 – 0.82)	**0.003**	0.56 (0.33 – 0.96)	**0.026**
	> 38 (f) / 55 (m) U/L	168	17.3 (12.7 – 20.9)	1		1	
TAT	≦ 91.61	21	14.5 (7.6 – 20.8)	1	0.15	-	-
	> 91.61	178	20.7 (15.9 – 25.1)	0.69 (0.42 – 1.14)		-	
IMAT	≦ 19.49	119	19.1 (15.6 – 24.8)	1	0.3	-	-
	> 19.49	79	17.4 (10.8 – 28.2)	0.75 (0.54 – 1.03)		-	
SAT	≦ 71.1 (f) / 71.16 (m)	55	15.6 (11.1 – 20.8)	1	0.34	-	-
	> 71.1 (f) / 71.16 (m)	143	22.2 (17.3 – 28.2)	0.84 (0.59 – 1.2)		-	
VAT	≦ 19.84 (f) / 72.95 (m)	116	16.0 (11.9 – 22.2)	1	0.12	-	-
	> 19.84 (f) / 72.95 (m)	82	25.7 (17.4 – 33.2)	0.77 (0.55 – 1.07)		-	
BONE	≦ 25.54 (f) / 29.75 (m)	69	19.2 (14.5 – 31.6)	1	0.39	-	
	> 25.54 (f) / 29.75 (m)	129	18.5 (13.3 – 24.4)	1.16 (0.83 – 1.63)		-	
MUSCLE	≦ 54.95 (f) / 85.95 (m)	91	15.0 (10.6 – 20.8)	1	**0.009**	-	-
	> 54.95 (f) / 85.95 (m)	107	24.4 (17.9 – 33.9)	0.65 (0.47 – 0.9)		-	
$\frac{\mathrm{MUSCLE}+\mathrm{VAT}}{\mathrm{BONE}}$	≦ 3.2 (f) / 4.66 (m)	86	13.1 (10.3 – 17.3)	1	**0.004**	-	**-**
	> 3.2 (f) / 4.66 (m)	112	24.8 (19.2 – 33.2)	0.62 (0.45 – 0.86)		-	
$\frac{\mathrm{MUSCLE}}{\mathrm{TAT}}$	≦ 0.57 (f) / 0.38 (m)	71	20.8 (12.6 – 30.4)	1	0.77	-	-
	> 0.57 (f) / 0.38 (m)	127	17.9 (13.1 – 22.9)	1.05 (0.75 – 1.47)		-	
$\frac{\mathrm{MUSCLE}}{\mathrm{BONE}}$	≦ 2.45 (f) / 2.48 (m)	69	12.0 (9.9 – 17.4)	1	**0.0001**	1	**0.008**
	> 2.45 (f) / 2.48 (m)	129	23.6 (18.5 – 34.0)	0.53 (0.38 – 0.74)		0.57 (0.38 – 0.86	
$\frac{\mathrm{MUSCLE}}{\mathrm{IMAT}}$	≦ 3.55 (f) / 3.74 (m)	64	15.1 (9.3 – 22.9)	1	**0.011**	-	-
	> 3.55 (f) / 3.74 (m)	134	20.7 (16.4 – 25.1)	0.65 (0.46 – 0.9)		-	
$\frac{\mathrm{MUSCLE}}{\mathrm{BONE}+\mathrm{IMAT}}$	≦ 1.69 (f) / 1.64 (m)	97	14.8 (11.2 – 22.2)	1	**0.002**	-	-
	> 1.69 (f) / 1.64 (m)	101	23.6 (17.5 – 34.0)	0.60 (0.43 – 0.82)		-	
$\frac{\mathrm{MUSCLE}+\mathrm{VAT}}{\mathrm{BONE}+\mathrm{IMAT}}$	≦ 1.96 (f) / 2.74 (m)	70	14.5 (9.4 – 22.2)	1	**0.009**	-	-
	> 1.96 (f) / 2.74 (m)	128	22.9 (17.5 – 30.4)	0.59 (0.43 – 0.83)		-	
$\frac{\mathrm{VAT}}{\mathrm{BONE}}$	≦ 2.29 (f) / 1.69 (m)	101	15.0 (12.2 – 18.5)	1	**0.018**	1	0.33
	> 2.29 (f) / 1.69 (m)	97	25.1 (18.5 – 33.2)	0.68 (0.49 – 0.94)		0.83 (0.57 – 1.21)	
$\frac{\mathrm{IMAT}}{\mathrm{BONE}}$	≦ 0.65	128	19.1 (16.04 – 26.3)	1	**0.027**	-	-
	> 0.65	80	15.9 (9.3 – 24.8)	1.44 (1.04 – 1.99)		-	

Abbreviations: ALAT, alanine aminotransferase; ALBI, albumin-bilirubin; ASAT, aspartate aminotransferase; BCLC, Barcelona Clinic Liver Cancer; CRP, C-reactive protein; GGT, gamma-glutamyl Transpeptidase; HCC, *Hepatocellular carcinoma*; IMAT, intramuscular adipose tissue; SAT, subcutaneous adipose tissue; TAT, Total adipose tissue; VAT, visceral adipose tissue. * BCLC stage A vs. B was not statistically significant in the UVA (p = 0.2493). BCLC stages A vs. C (p-value=0.008) and B vs. C (p-value=0.09) were statistically significant. In the multivariate analysis, only BCLC A vs. C (p = 0.003) and BCLC A vs. B (p = 0.0008) were found to be statistically significant. ** Liver enzymes were dichotomized at the gender specific upper level of normal for females (f) and males (m). For body composition parameters, sex-specific cutoffs were applied for females (f) and males (m), except for TAT, IMAT, and IMAT/B which did not show significant sex-specific differences.

**Fig. 3:**
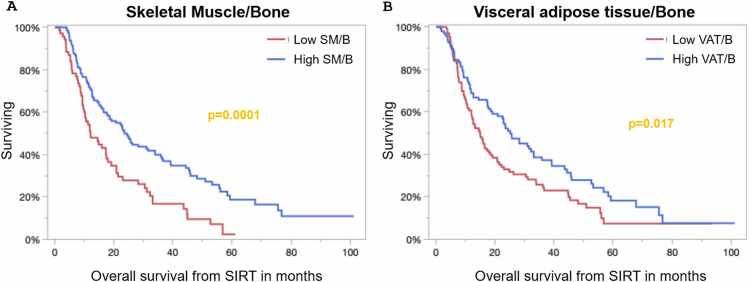
Overall survival graphs for body composition ratios A) skeletal muscle to bone ratio (SM/B) and B) visceral adipose tissue to bone ratio (VAT/B) are illustrated.

Considering body composition parameters, the sole predictor of overall survival for standalone parameters was greater muscle mass, with a median survival time of 24.4 months, compared to 15 months for patients with lower muscle mass (log-rank, p = 0.013). Furthermore, several indices and ratios based on the BCA parameters were evaluated. These results demonstrated the most effective survival discrimination between groups for the SM/B ratio, exhibiting a hazard ratio of 0.53 (95 % CI: 0.38–0.74) and a nearly doubled survival benefit, with a significantly prolonged median survival of nearly 12 months for patients with higher SM/B ratios (Fig. 3A). Furthermore, the inclusion of SM, IMAT, VAT, and B in several ratios and indices proved significant for survival discrimination, highlighting their relevance for survival prediction, as shown in Table 2. However, the SM/B ratio was the strongest prognostic factor, with no improvement in prognostic relevance from other body composition factors. Additionally, correlation analysis revealed a high correlation between the SM/B ratio and the other significant body composition factors identified in the univariate analysis (p ≤ 0.025), except for the VAT/B ratio (p = 0.14). Therefore, only SM/B, the body composition factor with the strongest hazard ratio, and VAT/B (Fig. 3B) were included in the multivariate analysis. Due to the overlap of the BCLC C cohort and portal vein thrombosis, only the BCLC stage was included in the multivariate analysis. Multivariate analysis revealed that BCLC stage was the most significant factor (p = 0.0009), followed by ASAT (p = 0.0027), SM/B ratio (p = 0.008), and GGT levels (p = 0.026) (Table 2).

Further subanalysis of the role of body composition markers for stratifying BCLC stage survival revealed no survival benefit in BCLC A patients, potentially due to the relatively low number of patients included. For BCLC B, high SM/B (HR: 0.4, 95 %CI: 0.25 – 0.64; p = 0.0001), SM/(B+IMAT) (HR: 0.6, 95 %CI: 0.4 – 0.94; p = 0.005), (SM+VAT)/(B+IMAT) (HR: 0.56, 95 % CI: 0.36 – 0.86; p = 0.008), (SM+VAT)/B (HR: 0.6, 95 % CI: 0.41 – 0.97), and VAT/B (HR: 0.52, 95 %CI: 0.34 – 0.8; p = 0.003) were statistically significant in the univariate analysis. For BCLC C, only high SM/(B+IMAT) (HR: 0.4, 95 %CI: 0.23 – 0.77, p = 0.005) and (SM+VAT)/(B+IMAT) (HR: 0.4, 95 %CI: 0.22 – 0.76, p = 0.005) were statistically significant.

Notably, muscle mass, visceral adipose tissue, and other body composition markers did not show statistically significant differences between BCLC stages when subjected to ANOVA testing. In addition, VAT and IMAT were highly positively correlated with each other (p < 0.0001). Six-month follow-up imaging in 143 patients showed median reductions in SM/B to 98.6 % and VAT/B to 88.9 % of their baseline values. However, these changes were not significantly associated with overall survival.

### 3.3 Response assessment

The median time to progression was 7.2 months (95 % CI: 6.1–7.9). The best achieved response was complete remission in 3.6 %, partial response in 34.9 %, stable disease in 29.7 %, and progressive disease in 31.8 % of patients. Regarding BCA parameters, patients exhibiting higher SM/B values demonstrated a considerably higher disease control rate at the first imaging follow-up after 3 months, with a percentage of 74.4 %, in contrast to patients with low values, which reached 54.0 % (p = 0.017). No statistically significant difference was observed for VAT/B (p = 0.27). In terms of time to progression, a lower hazard ratio and longer TTP was observed only for the following parameters from Table 2: patients aged ≤ 70 years (HR: 0.59, 95 % CI: 0.4 – 0.85, p = 0.005; TTP: 8.4 vs. 6.1 months), for patients with no portal vein thrombosis (HR: 0.54, 95 % CI: 0.34 – 0.86, p = 0.009; TTP: 7.3 vs. 4.4 months) and for patients with higher SM/B ratios (HR: 0.68, 95 % CI: 0.46 – 0.998, p = 0.049; TTP: 7.8 vs. 6.3 months). However, in the multivariate analysis, only younger age (HR: 0.63, 95 % CI: 0.41 – 0.96; p = 0.03) and absence of portal vein thrombosis (HR: 0.51, 95 % CI: 0.32 – 0.81, p = 0.004) whereas higer SM/B ratio did not remain significant (HR: 0.84, 95 % CI: 0.54 – 1.31, p = 0.45).

Notably, in the BCLC B subgroup, SM/B demonstrated a significantly lower hazard ratio of 0.42 (95 % CI: 0.25 – 0.73; p = 0.002), with a median time to progression of 11.3 (6.9 – 16.2 vs. 6 months (95 % CI: 3.9 – 7.3). However, when accounting for age, this finding lost statistical significance.

## 4 Discussion

Radioembolization remains a crucial component of the effective multidisciplinary management of patients with *Hepatocellular carcinoma* (HCC) [26], [27]. In clinical practice, many prognostic markers have been established, most of which are based on clinical information, laboratory markers, and conventional radiological tumor characteristics [27]. Nevertheless, it remains challenging to determine whether patients will derive substantial benefits from radioembolization. Therefore, the use of additional prognostic markers could improve the estimation of benefits and help manage expectations for patients and their relatives.

A comprehensive meta-analysis revealed a substantial impact of sarcopenia on the prognosis of *Hepatocellular carcinoma* (HCC), irrespective of the treatment modality, although data on radioembolization included in this study were very limited [6]. Despite substantial evidence supporting the potential benefits of body composition markers, including CT-based sarcopenia, their utilization in clinical routines remains limited [3], [4], [16], [17], [18], [19]. Nam et al. reported a significant survival benefit for patients with higher sex-specific skeletal muscle index, defined as the skeletal muscle area (cm²) divided by body height in squared meters at the level of the third lumbar vertebra. The analysis revealed that the survival benefit associated with a normal skeletal muscle index was significantly higher at 35.3 months than at 21.1 months for those with a low skeletal muscle index [4]. Surov et al. conducted an investigation into patients from the SORAMIC trial (patients treated with sorafenib plus or minus radioembolization) at the L3 level.

Nevertheless, the study revealed that markers of the area, with and without normalization by body height for muscle, VAT, TAT, SAT, and IMAT, were not prognostically relevant [16]. Despite undergoing radioembolization, the underlying causes of these negative findings remain incompletely elucidated. However, given the median overall survival of only 9.9 months and the absence of therapeutic benefit in either cohort, it is plausible that the prognostic significance of body composition markers may be limited in patients with a particularly poor prognosis. In addition, the employed ratios incorporating bone volume have not been used in this study. As bone volume remains relatively constant despite changes in fat or muscle volume, this marker may be helpful as an additional internal normalization parameter to achieve significance. Notwithstanding this adverse finding, a subsequent post-hoc radiomics analysis demonstrated the capacity of the radiomics features of the body composition compartments mentioned above to predict survival periods exceeding one year, indicating the relevance of body composition beyond tissue volume [28].

Despite the heterogeneity of the findings and the differing methodologies used for body composition assessment and treatment strategies, the present results corroborate the hypothesis that body composition parameters, particularly muscle mass, play a significant role in predicting a greater probability of survival over an extended period in patients treated with radioembolization. While the precise mechanisms governing this relationship remain unclear, one plausible interpretation is that the immune response exerts a significant influence on the outcomes of transarterial radioembolization. Valerie et al. established a significant correlation between immune cell activation and a sustained response to radioembolization. Their investigation revealed that the pronounced activation of CD8 + T lymphocytes within both the tumor microenvironment and systemic milieu was markedly correlated with favorable outcomes following radioembolization [29]. Additionally, it is worth noting that skeletal muscle, in its capacity as an endocrine organ, has been shown to secrete cytokines. These cytokines have been identified as critical regulators of immunity and play a pivotal role in the beneficial modulation of the immune response [30]. Considering the intimate connection between sarcopenia and chronic systemic inflammation with impaired antitumor immune response, this observation may explain the association between muscle mass and prolonged survival in patients treated with radioembolization [30], [31], [32]. The findings of this study provide preliminary evidence suggesting that patients with higher SM/B ratios may experience higher disease control rates and longer time to progression, corroborating the previously documented beneficial effects of muscle volume. However, given the significant correlation between age and muscle mass and the fact that only age and portal vein thrombosis remained significant in the multivariate analysis of time to progression, the value of muscle mass may be subordinate.

In contrast to skeletal muscle volume, the roles of visceral adiposity and myosteatosis in patients with *Hepatocellular carcinoma* (HCC) remain ambiguous. A high visceral adipose tissue (VAT) index has been associated with shortened survival in patients treated with transarterial chemoembolization (TACE) [18], [33] or hepatic resection [33]. In contrast, a higher VAT has been associated with improved outcomes in patients receiving sorafenib [12] and immunotherapy [14].

Although Fujiwara et al. demonstrated the prognostic role of intramuscular fat in patients with HCC in terms of survival outcomes [8], the prognostic relevance of intramuscular fat in patients treated with locoregional therapies, such as TACE or sorafenib, with or without radioembolization, has not been proven [16], [34].

In the present study, VAT/B, IMAT/B, and various combinations of VAT and IMAT with SM/B showed significance in the univariate analysis; however, they were not superior or independent compared to the SM/B ratio itself, thereby demonstrating a minimal overall additive prognostic benefit of these factors in patients treated with radioembolization. Ebadi et al. discovered that patients with a higher Hounsfield score of less than −85 Hounsfield units exhibited a significantly prolonged survival period [17]. Elevated portal vein pressure, which increases the proportion of fluid within the VAT and thus increases the Hounsfield units, is a widely accepted marker of poor survival in patients with liver cirrhosis [35]. However, it is worth noting that many other factors can influence the radiodensity of visceral adipose tissue. These include various fat compositions, such as phospholipids and waxy lipids, which are more radiodense [36]. This highlights the possibility that the quality and composition of visceral adipose tissue, rather than the mass alone, may play a crucial role in its prognostic value.

Recent meta-analytical data reveals a geographic disparity in the burden of myosteatosis: while the pooled prevalence is 45 % in Asian cohorts, it rises significantly to 69 % in non-Asian cohorts. Furthermore, an analysis of six studies assessed the prognostic value of this condition in patients with HCC; the results demonstrated a significant association with reduced survival, yielding a pooled Relative Risk of 1.35 (95 % CI: 1.13–1.62, p < 0.01) [15]. In this study, higher IMAT, whether normalized by bone (HR:1.44, 95 % CI: 1.04–1.99, p = 0.027) or as a fraction to muscle (HR: 1.54, 95 % CI: 1.11–2.17, p = 0.011), demonstrated a comparable mortality risk ratio in our study cohort. Further research is required to elucidate the relationship with other factors affecting IMAT such as diabetes, and to determine the potential efficacy of interventions such as physical exercise and nutritional interventions [37].

Beyond their ability to stratify survival risks in the overall patient population, the analysis emphasized the specific clinical utility of BCA parameters in BCLC B patients—a subgroup known for its heterogeneity and variable prognostic outcomes. Notably, in the BCLC C subset analysis, the amount of intramuscular fat in the SM/B ratio appeared to be a relevant factor in patients with advanced HCC disease indicating that the predictive value of myosteatosis evolves as the disease progresses to advanced stages.

The observation that subcutaneous adipose tissue lacks prognostic significance aligns with a pooled analysis of twelve studies [38]. That meta-analysis found no significant association between subcutaneous fat volume and outcomes in HCC patients receiving surgery, locoregional therapy, or tyrosine kinase inhibitors (Sorafenib/Brivanib). However, the predictive value of this fat depot appears to be treatment-dependent. In contrast to standard therapies, recent evidence identifies subcutaneous adipose tissue volume as an independent prognostic factor specifically in patients treated with immune checkpoint inhibitors

. In the analysis of body composition alterations at six weeks, no significant association with overall survival was observed. In the literature regarding conventional TACE, contradictory findings have been reported: one study identified skeletal muscle and visceral adipose tissue loss as prognostically relevant for shorter survival [39], while another study did not find a statistically significant difference [40]. Overall, further research is warranted to elucidate the potential impact of body composition changes on clinical outcomes in greater depth.

However, whether nutritional and exercise interventions benefit patients with unfavorable body composition parameters remains to be elucidated. Thought ahead, as many patients with liver disease undergo CT imaging for HCC screening, using the fully automated body composition analysis tool may allow for easy and standardized monitoring of sarcopenia, which will in turn allow for intensified early nutritional and exercise interventions with their possible potential benefits, as recommended by the current ACG clinical guidelines on malnutrition and nutritional recommendations in liver disease [41]. This may ultimately improve treatment outcomes in cases where radioembolization is warranted for HCC treatment.

This study had several limitations. First, the data were obtained from a single institution, and only a relatively low proportion of patients treated had suitable pretherapeutic CT images available in the institutional picture archiving and communication system, which may have introduced selection bias. Furthermore, the quantification method employed in this study differs significantly from those employed in previous studies and from the published cutoffs and normal ranges in healthy individuals, which limits its comparability with the literature and transferability. Therefore, the selected cutoffs for each sex in this study cohort, without any healthy control group for comparison and standardization, may require further refinement and validation of the ideal cutoff values. Additionally, the radiation dosage scheme applied was not based on personalized dosimetry, as is now customary, which may limit its transferability to the current standard of care for radioembolization. The patients included in this study were deemed unsuitable for curative treatment and unsuitable for systemic therapy by the interdisciplinary tumor board at the time. The advent of novel systemic therapies has increased the likelihood that a considerable proportion of patients included in this study would now preferentially be treated with systemic therapies instead of radioembolization, which is in accordance with the prevailing EASL guidelines [2]. This development may further limit the generalizability and applicability of the study's findings in clinical practice, underscoring the need for further investigation. Moreover, the presence of comorbidities, which have the capacity to act as potential confounders, particularly in the context of age-related survival and impact on body composition parameter outcomes, was not taken into account. This necessitates further investigation.

## 5 Conclusion

The integration of fully automated, whole-abdomen CT-based body composition analysis offers a promising tool for identifying patients who will derive the greatest benefit from radioembolization. Notably, the skeletal muscle/bone ratio emerged as the most significant metric, serving as an independent prognostic factor for overall survival. Furthermore, these parameters enable a more granular stratification of BCLC B and C patients, ultimately supporting a shift toward more personalized HCC management strategies.

## Abbreviations

ASAT: Aspartate Aminotransferase, B: Bone Volume, BCA: Body Composition Analysis, BCLC: Barcelona Clinic Liver Cancer, CI: Confidence Interval, CT: Computed Tomography, GGT: Gamma-Glutamyl Transaminase, HCC: *Hepatocellular carcinoma*, HR: Hazard Ratio, HU: Hounsfied Unit, IMAT: Intra- and Intermuscular Adipose Tissue Volume, IQR: Interquartile Range, MAA: Macroaggregated Albumin, mRECIST: Modified Response Evaluation Criteria in Solid Tumors, MVA: Multivariate Analysis, OS: Overall Survival, SPECT: Single-Photon Emission Computed Tomography, SM: Skeletal Muscle (Volume), SAT: Subcutaneous Adipose Tissue Volume, TACE: Transarterial Chemoembolization, TAT: Total Adipose Tissue Volume, TTP: Time to Progression, UVA: Univariate Analysis, VAT: Visceral Adipose Tissue Volume

## Ethical statement

Institutional review board approval was obtained for this single-center cohort study and informed consent was waived due to its retrospective nature (21–10097-BO and 13–5325-BO).

## CRediT authorship contribution statement

**Anneke Ketelsen:** Writing – review & editing, Formal analysis, Data curation. **Steinberg-Vorhoff Hannah Luisa:** Writing – original draft, Visualization, Validation, Supervision, Software, Project administration, Formal analysis, Data curation, Conceptualization. **Theysohn Jens M.:** Writing – review & editing, Visualization, Resources, Investigation, Formal analysis, Conceptualization. **Tabea Schuch:** Writing – review & editing, Formal analysis, Data curation. **Johannes Haubold:** Writing – review & editing, Validation, Methodology, Investigation. **Benedikt M. Schaarschmidt:** Writing – review & editing, Resources, Investigation, Formal analysis, Conceptualization. **Matthias Jeschke:** Writing – review & editing, Validation, Methodology, Investigation, Data curation, Conceptualization. **Farroch Vahidi Noghani:** Writing – review & editing, Methodology, Investigation, Formal analysis, Data curation. **Johannes M. Ludwig:** Writing – original draft, Visualization, Validation, Supervision, Project administration, Methodology, Investigation, Formal analysis, Data curation, Conceptualization. **Leonie Jochheim:** Writing – original draft, Validation, Supervision, Methodology, Investigation, Formal analysis, Conceptualization.

## Funding

This research did not receive any specific grant from funding agencies in the public, commercial, or not-for-profit sectors. Open Access funding enabled and organized by Projekt DEAL.

## Declaration of Competing Interest

The authors declare the following financial interests/personal relationships which may be considered as potential competing interests: Benedikt Schaarschmidt reports a relationship with German Research Foundation that includes: funding grants. Benedikt Schaarschmidt reports a relationship with PharmaCept GmbH that includes: funding grants. Benedikt Schaarschmidt reports a relationship with Else Kroner-Fresenius Foundation that includes: funding grants. Benedikt Schaarschmidt reports a relationship with AstraZeneca GmbH that includes: speaking and lecture fees. Benedikt Schaarschmidt reports a relationship with Bayer AG that includes: funding grants. Jens Theysohn reports a relationship with Sirtex that includes: consulting or advisory. Jens Theysohn reports a relationship with Boston Scientific that includes: consulting or advisory. Benedikt Schaarschmidt reports a relationship with Boston Scientific that includes: consulting or advisory. Hannah Steinberg-Vorhoff reports a relationship with Boston Scientific that includes: consulting or advisory. If there are other authors, they declare that they have no known competing financial interests or personal relationships that could have appeared to influence the work reported in this paper.
