# Supplementary material for: Muscle and fat matter: Automated CT-based body composition analysis predicts survival in Hepatocellular carcinoma patients undergoing radioembolization
Source: Eur J Radiol Open. 2026 Jan 19;16:100721. doi: 10.1016/j.ejro.2025.100721 (PMC12856858; doi:10.1016/j.ejro.2025.100721)
Supplement: Supplementary file 1 — Supplementary material [file mmc1.docx]

Table S1:

Median values and interquartile ranges (IQR) for body composition markers for the entire cohort, female and male patients.

| Parameter | Overall (IQR) | Female (IQR) | Male (IQR) | p-value |
| --- | --- | --- | --- | --- |
| BONE | 30.23 (27.09-33.29) | 25.54 (23.35-27.59) | 31.55 (29.2-34.05) | <0.0001 |
| MUSCLE | 81.93 (65.69-91.55) | 62.87 (52.91-70.2) | 85.95 (74.59-92.63) | <0.0001 |
| TAT | 173.41 (134.17-210.43) | 189.4 (135.46-208.56) | 171.82 (134.15-213.37) | 0.79 |
| IMAT | 16.85 (13.27-24.34) | 16.47 (12.49-20.42) | 16.94 (13.37-25.09) | 0.06 |
| SAT | 91.39 (68.02-130.57) | 118.04 (85.56-146.71) | 85.09 (67.56-121.01) | 0.003 |
| VAT | 54.13 (38.17-71.96) | 37.76 (21.48-52.53) | 59.13 (43.98-76.88) | <0.0001 |
| MUSCLE + VAT / BONE | 4.53 (3.82-5.11) | 3.93 (3.51-4.5) | 4.66 (3.99-5.18) | <0.0001 |
| MUSCLE/TAT | 0.47 (0.35-0.59) | 0.35 (0.29-0.46) | 0.5 (0.4-0.62) | 0.0004 |
| MUSCLE/BONE | 2.61 (2.33-2.99) | 2.45 (2.18-2.64) | 2.74 (2.39-3.06) | 0.002 |
| MUSCLE/IMAT | 4.51 (3.15-5.85) | 3.7 (2.99-4.64) | 4.87 (3.43-6.03) | 0.003 |
| MUSCLE/(BONE+IMAT) | 1.67 (1.37-1.91) | 1.47 (1.31-1.63) | 1.72 (1.4-1.98) | 0.0003 |
| (MUSCLE+VAT)/(BONE+IMAT) | 2.75 (2.35-3.16) | 2.31 (1.99-2.58) | 2.85 (2.44-3.24) | <0.0001 |
| VAT/BONE | 1.81 (1.4-2.28) | 1.61 (0.93-2.03) | 1.85 (1.48-2.39) | 0.0013 |
| IMAT/BONE | 0.58 (0.46–0.79) | 0.65 (0.53–0.83) | 0.56 (0.45–0.77) | 0.01 |

The median values of body composition parameters for the entire cohort and for female and male patients are shown, along with the interquartile range (IQR). The significance of the observed differences between females and males was determined through the implementation of the Student's t-test following confirmation of normal distribution.

Table S2: Calculated cutoffs for each body composition parameter for the entire study cohort, female and male patients respectively.

| Parameter | Overall cutoff | Female cutoff | Male cutoff |
| --- | --- | --- | --- |
| BONE | 26.02 | 25.54 | 29.75 |
| MUSCLE | 74.98 | 54.95 | 85.95 |
| TAT | 91.96 | 106.01 | 194.55 |
| IMAT | 19.49 | 11.07 | 19.98 |
| SAT | 74.19 | 71.1 | 71.16 |
| VAT | 89.92 | 19.89 | 72.95 |
| MUSCLE + VAT / BONE | 3.39 | 3.2 | 4.66 |
| MUSCLE/TAT | 0.42 | 0.57 | 0.38 |
| MUSCLE/BONE | 2.39 | 2.45 | 2.48 |
| MUSCLE/IMAT | 3.98 | 3.55 | 3.74 |
| MUSCLE/(BONE+IMAT) | 1.54 | 1.69 | 1.64 |
| (MUSCLE+VAT)/(BONE+IMAT) | 2.75 | 1.96 | 2.74 |
| VAT/BONE | 1.65 | 2.29 | 1.69 |
| IMAT/BONE | 0.65 | 0.65 | 0.67 |

Body composition parameters, indices and ratios were dichotomized utilising a maximally selected log-rank approach, the 10th – 90th percentile values (in 10% steps) for each parameter were examined, and the cut-point that yielded the smallest two-sided log-rank p-value between the "low" (≤ cut-off) and "high" (> cut-off) groups was selected. In case of statistically significant sex-related difference of body composition parameters, a gender specific cutoff was applied.
